# Exosomal miR-141-3p regulates osteoblast activity to promote the osteoblastic metastasis of prostate cancer

**DOI:** 10.18632/oncotarget.22014

**Published:** 2017-10-24

**Authors:** Yun Ye, Su-Liang Li, Yue-Yun Ma, Yan-Jun Diao, Liu Yang, Ming-Quan Su, Zhuo Li, Yang Ji, Juan Wang, Lin Lei, Wei-Xiao Fan, La-Xiu Li, Yi Xu, Xiao-Ke Hao

**Affiliations:** ^1^ Department of Laboratory Medicine, Xijing Hospital, Fourth Military Medical University, Xi’an, Shaanxi 710032, China; ^2^ Department of Clinical Laboratory, The First Affiliated Hospital of Xi’an Medical University, Xi’an, Shaanxi 710077, China; ^3^ Department of Radiology, The First Affiliated Hospital of Xi’an Medical University, Xi’an, Shaanxi 710077, China

**Keywords:** exosome, miR-141-3p, DLC1, p38MAPK, osteoblast activity

## Abstract

Exosomes from cancer cells, which contain microRNA and reach metastasis loci prior to cancer cells, stimulate the formation of a metastatic microenvironment. Previous studies have shown that exosomal miR-141-3p is associated with metastatic prostate cancer (PCa). However, the role and regulatory mechanism of miR-141-3p in the microenvironment of bone metastases require further study. In this study, we performed a series of experiments *in vivo* and *in vitro* to determine whether exosomal miR-141-3p from MDA PCa 2b cells regulates osteoblast activity to promote osteoblastic metastasis. We demonstrate that extracts obtained from cell culture supernatants contained exosomes and that miR-141-3p levels were significantly higher in MDA PCa 2b cell exosomes. Via confocal imaging, numerous MDA PCa 2b exosomes were observed to enter osteoblasts, and miR-141-3p was transferred to osteoblasts through MDA PCa 2b exosomes *in vitro*. Exosomal miR-141-3p from MDA PCa 2b promoted osteoblast activity and increased osteoprotegerin OPG expression. miR-141-3p suppressed the protein levels of the target gene DLC1, indicating its functional significance in activating the p38MAPK pathway. In animal experiments, exosomal miR-141-3p had bone-target specificity and promoted osteoblast activity. Mice injected with miR-141-3p-mimics exosomes developed apparent osteoblastic bone metastasis. Exosomal miR-141-3p from MDA PCa 2b cells promoted osteoblast activity and regulated the microenvironment of bone metastases, which plays an important role in the formation of bone metastases and osteogenesis damage in PCa. Clarifying the specific mechanism of bone metastasis will help generate new possibilities for the treatment of PCa.

## INTRODUCTION

Prostate cancer (PCa) is a common malignant tumour of the male urogenital system, the second leading cause of cancer mortality in men worldwide and a significant cause of death in elderly men [1, 2]. Bone is the most common site of PCa metastasis; approximately 90% of patients with metastatic disease will develop bone metastases that are predominantly of the osteoblastic bone-forming type [3, 4]. Studies have shown that PCa cells interact with the microenvironment of bone metastases through a variety of cytokines, extracellular matrix components and intercellular signalling networks, which play an important role in the occurrence and development of bone metastases [5, 6]. Therefore, clarifying the mechanism by which cancer cells interact with the bone metastatic microenvironment to establish PCa bone metastases is essential for developing disease prevention and control strategies and improving disease prognosis.

Exosomes are extracellular vesicles of endocytic origin that range in size from 30-120 nm and are released under physiological and pathological conditions [7]. The content of exosomes reflects the cell of origin and includes lipids, proteins, mRNAs and microRNAs (miRNAs), which are transferred from donor cells to target cells. As a form of intercellular vesicular transport, exosome-mediated intercellular communication and gene rearrangement of target cells participate in a variety of pathological processes [8]. Cancer cells have been shown to release a variety of exosomes that contain miRNAs and reach metastatic loci prior to cancer cells, stimulating the transfer of the cancer microenvironment and thereby promoting tumour metastasis [9]. Intercellular information transmission mediated by exosomes may represent a new mode of communication between the tumour and its microenvironment [10]. Clinical trials and animal studies suggest that PCa is associated with miR-141-3p levels and that metastatic PCa exhibits significantly higher miR-141-3p levels than localized PCa [11, 12]. Our previous work demonstrated that exosomal miR-141-3p is upregulated in the serum of metastatic PCa patients, which is related to bone metastasis [13]. However, the role and regulatory mechanism of miR-141-3p in the bone metastasis microenvironment remains poorly understood.

In this study, we found that miR-141-3p released from PCa cells could be transferred to osteoblasts and promote osteoblast activity, which was conducive to the formation of a bone-metastasis microenvironment. Upon uptake, miR-141-3p reduced the protein levels of its target gene DLC1 and activated p38MAPK signalling, which increased the expression of osteoblast osteoprotegerin *OPG* and further promoted bone formation. We present *in vivo* evidence to demonstrate that exosomal miR-141-3p from MDA PCa 2b cells had bone-target specificity and regulated the bone microenvironment to promote osteoblastic metastasis.

## RESULTS

### Characterization of exosomes and miR-141-3p expression

**Figure 1:**
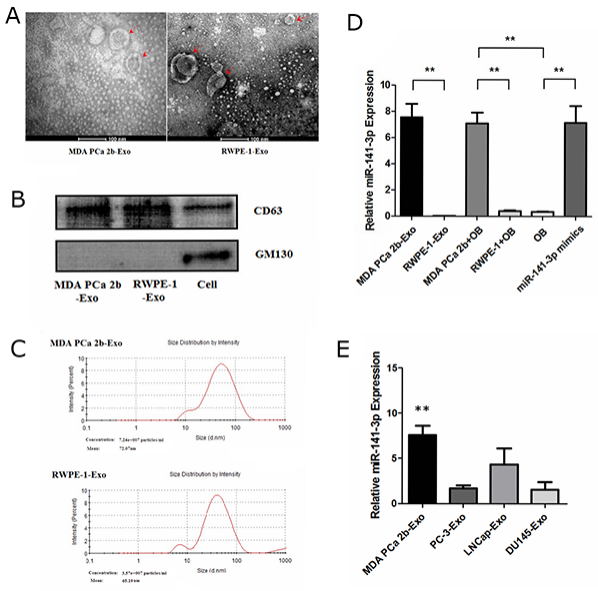
Characterization of exosomes and expression of miR-141-3p **(A)** Morphological analysis via transmission electron microscopy. **(B)** Western blotting of exosomal membrane markers. **(C)** The size distribution of exosomes. **(D)** Real-time PCR analysis of exosomal miR-141-3p expression. **(E)** The level of miR-141-3p in exosomes from metastatic prostate cancer cell lines. Note: the data represent the mean ± SD. ^**^P < 0.01.

We incubated MDA PCa 2b cells (a PCa bone-metastasis cell line) and RWPE-1 cells (human prostatic epithelial cells) in exosome-free medium generated from exosome-free FBS. Exosomes were isolated from cell culture medium collected after 48 h by differential centrifugation as described previously [14]. To examine the size distribution and morphology of the isolated exosomes, the exosome pellets were resuspended in PBS and then examined by nanoparticle tracking analysis and transmission electron microscopy. The isolated exosomes appeared as uniformly round or oval membrane vesicles (Figure 1A) with sizes within the characteristic diameter range of 30-120 nm (Figure 1C). The shape and size of the exosomes were consistent with reported exosome characteristics [15]. To further characterize the isolated exosomes, we used the exosomal protein marker CD63 and the cis-Golgi marker GM130, which is only present in cell lysates. As shown in Figure 1B, the isolated exosomes were positive for the exosomal marker CD63 and negative for the cis-Golgi marker GM130. These results confirmed that the vesicles isolated from the conditioned media were exosomes based on their morphology, size and marker protein expression.

To search for candidate miRNAs that were potentially involved in mediating the bone microenvironment in PCa, we selected 10 miRNAs that were previously reported to be involved in metastases [16, 17, 18] (Supplementary Table 1). We examined the expression of those miRNAs in exosomes from the cell supernatant using real-time PCR. Compared with exosomes from normal RWPE-1 prostate epithelial cells, in exosomes from MDA PCa 2b cells, only the levels of miR-141-3p but not the other examined miRNAs were increased significantly (Figure 1D) (Supplementary Figure 1). To investigate whether the exosomes from metastatic PCa cells contain a high amount of miR-141-3p, we examined miR-141-3p expression in exosomes from the following cell lines: MDA PCa 2b; PC-3; DU-145; and LNCap. The results show that the levels of miR-141-3p in exosomes from MDA PCa 2b cells were significantly higher than those in the other cell lines (Figure 1E).

### Transfer of miR-141-3p from donor cells to recipient cells through exosomes

**Figure 2:**
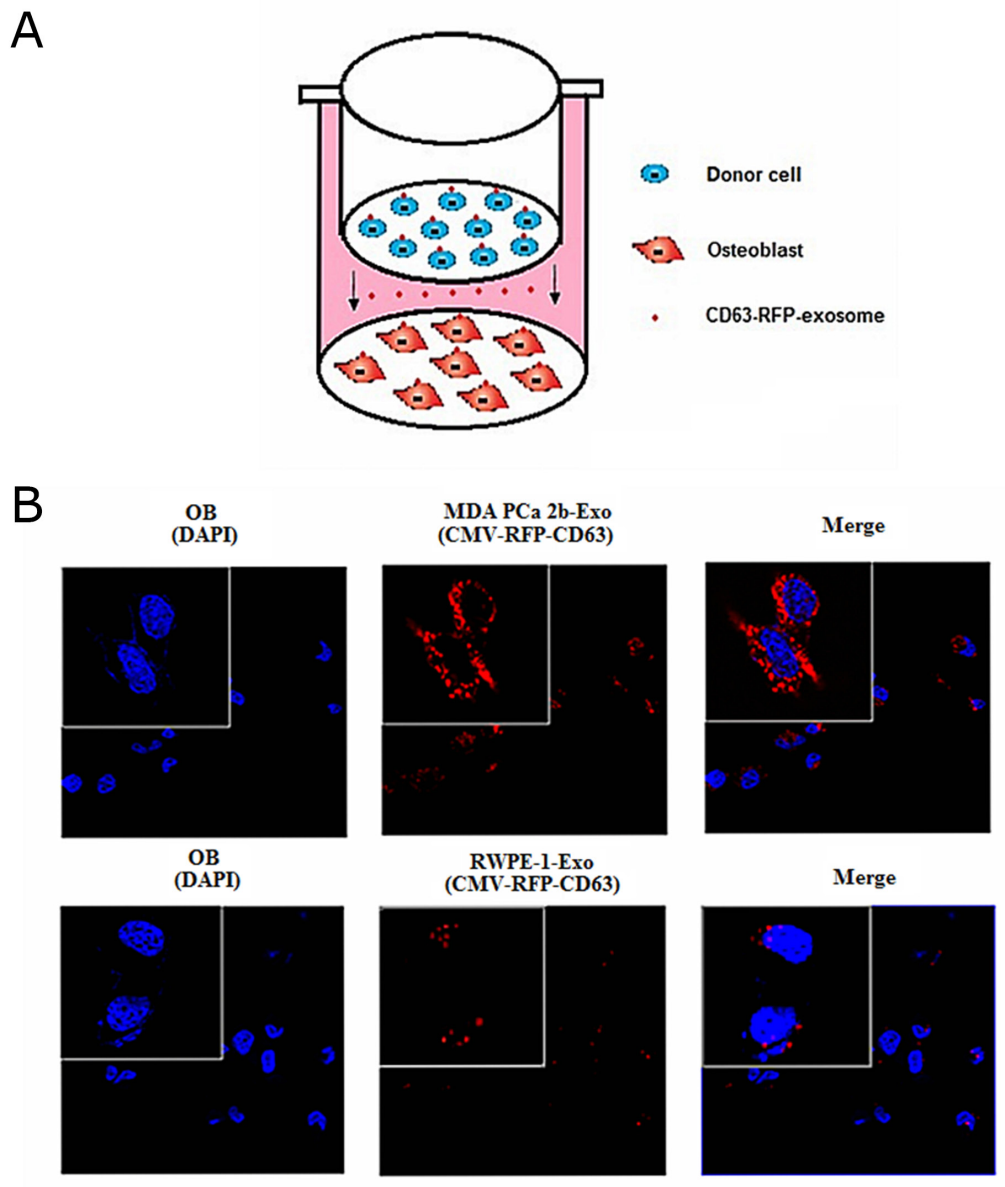
Transfer of MDA PCa 2b cells -derived exosomal miR-141-3p to osteoblasts **(A)** Schematic diagram illustrating the design of co-culture experiments. **(B)** Representative confocal images of CD63-RFP-exosome by donor cells at 72 h after co-culture.

We first verified whether exosomal miR-141-3p was transferred from donor cells to osteoblasts. We co-cultured either MDA PCa 2b or RWPE-1 cells with osteoblasts in a transwell system with a 0.4-mm pore polyethylene terephthalate (PET) membrane that allows the transfer of exosomes [19] (Figure 2A). We constructed a lentiviral vector system containing cytomegalovirus (CMV)-driven red fluorescence protein (RFP)-tagged CD63 (CMV-RFP-CD63) to label the exosomes derived from MDA PCa 2b and RWPE-1 cells. Numerous RFP+ particles were present within the osteoblasts after 72 h of co-culture with MDA PCa 2b cells, as observed by confocal imaging (Figure 2B). Real-time PCR was applied to confirm whether the MDA PCa 2b-derived exosomal miR-141-3p was responsible for the elevated miR-141-3p in osteoblasts. The miR-141-3p level in osteoblasts co-cultured with MDA PCa 2b cells was significantly higher than that in osteoblasts alone or in osteoblasts co-cultured with RWPE-1 cells at 72 h after co-culture. These results indicate that miR-141-3p was transferred to osteoblasts via exosomes *in vitro* (Figure 1D). Based on these results, we next investigated the role of this transferred miR-141-3p in osteoblasts.

### miR-141-3p promote the activity of osteoblasts

**Figure 3:**
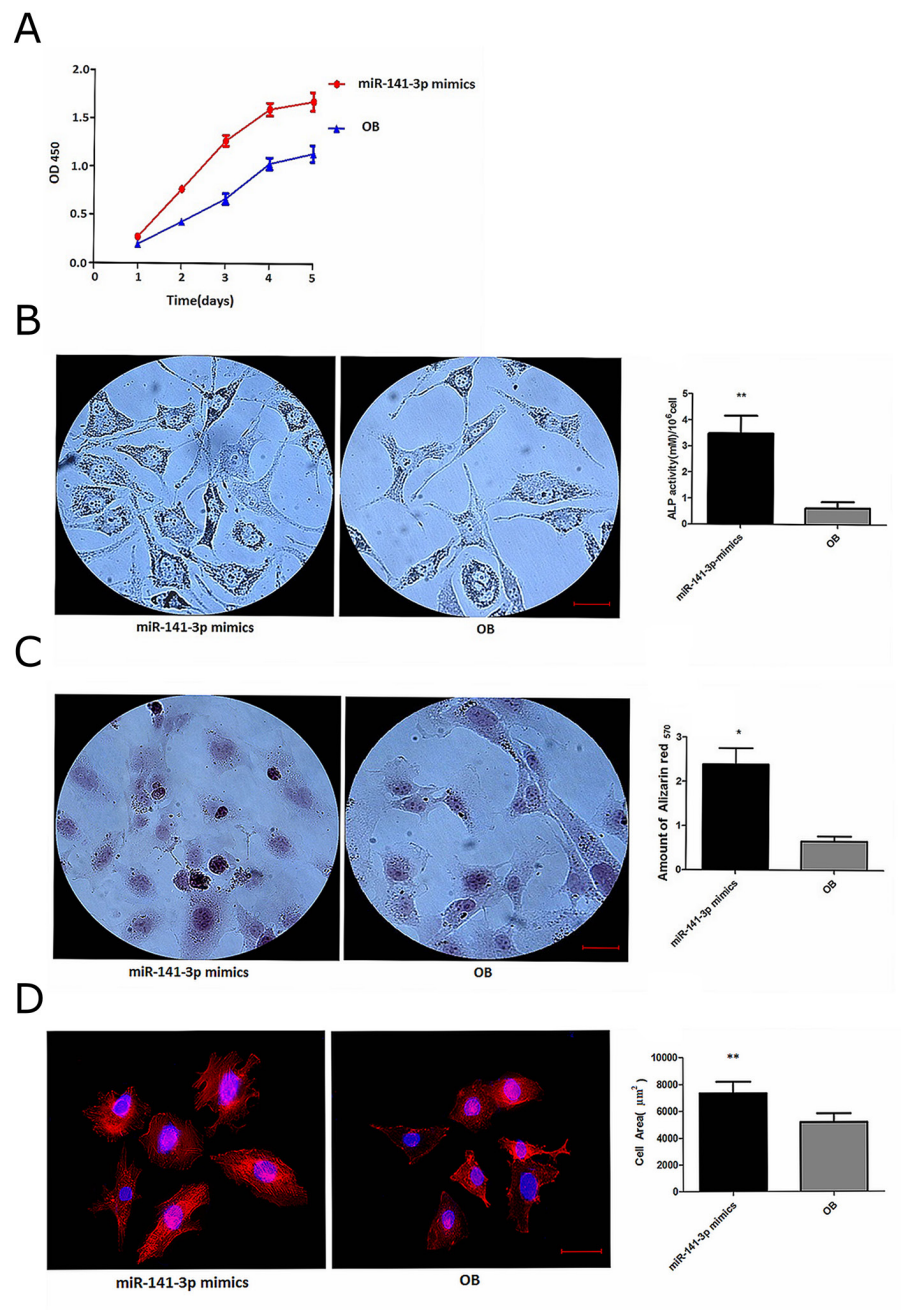
miR-141-3p promotes osteoblast activity **(A)** Osteoblast proliferation. **(B)** Alkaline phosphatase activity in osteoblasts. **(C)** The level of extracellular matrix mineralization in osteoblasts. **(D)** Osteoblast adhesion. Scale bars, 25 μm.

Cancer cell exosomes drive miRNA transfer and further affect cell status in metastasis sites, which is closely related to the formation of tumour metastasis microenvironments [20]. To determine whether exosomal miR-141-3p affects osteoblasts, we transfected osteoblasts with miR-141-3p mimics to alter the level of miR-141-3p. Real-time PCR analysis revealed dramatically higher osteoblast miR-141-3p levels after 5 days in osteoblasts transfected with miR-141-3p mimics compared with non-transfected osteoblasts (Figure 1D). We found that osteoblast activity was significantly affected by changes in miR-141-3p levels. CCK-8 test results showed that miR-141-3p significantly promoted osteoblast proliferation (Figure 3A). Alkaline phosphatase activity and extracellular matrix mineralization are important indicators of osteoblast differentiation [21, 22]. Alkaline phosphatase staining showed that the vast majority of osteoblasts contained black-grey particles after miR-141-3p mimic transfection for 5 days compared with non-transfected cells, with significant differences in the number of positive cells and the level of alkaline phosphatase activity (Figure 3B). We used alizarin red staining of osteoblast-secreted calcium nodules to assess extracellular matrix mineralization, and we quantitatively analysed the level of extracellular matrix mineralization by measuring the absorbance of cetylpyridinium chloride (CPC) in combination with alizarin red staining of calcium nodules. The results showed that the level and quantity of calcium nodule deposition were significantly higher than the level and quantity in non-transfected cells after 5 days of transfection with miR-141-3p mimics (Figure 3C). We then observed rhodamine-phalloidin and DAPI double-staining of actin microfilaments and nuclei, respectively, by confocal microscopy to evaluate osteoblast adhesion. Osteoblasts showed good morphology and obvious actin microfilaments, and the cell area was significantly increased after miR-141-3p mimic transfection for 5 days (Figure 3D). Taken together, these results indicate that miR-141-3p promotes osteoblast activity *in vitro*.

### miR-141-3p and OPG/RANKL expression

**Figure 4:**
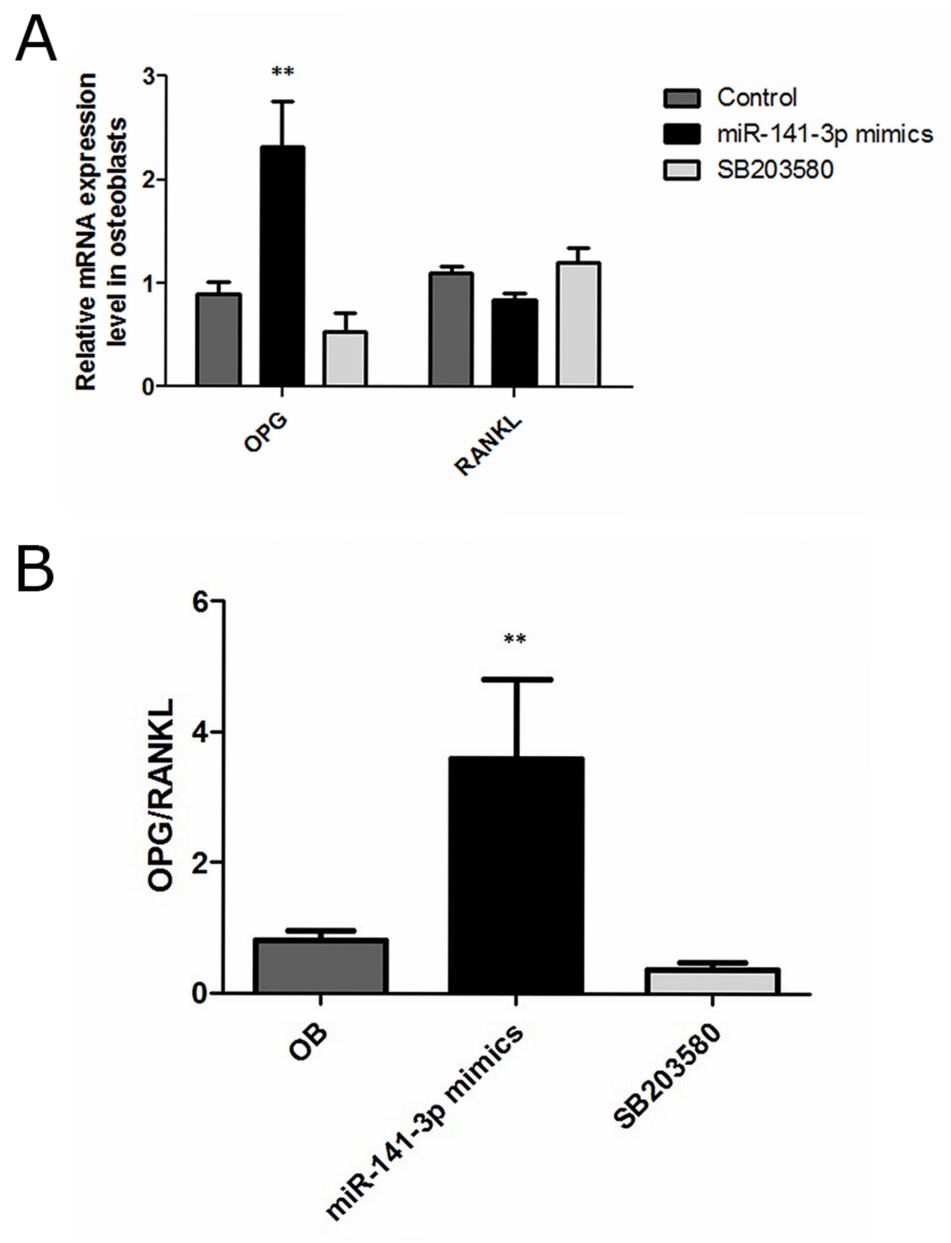
OPG, RANKL expression in osteoblasts **(A)** OPG and RANKL expression. **(B)** OPG/RANKL in osteoblasts.

Previous studies have shown that OPG and RANKL expression is developmentally regulated by osteoblasts [23]. OPG is a member of the tumour necrosis factor receptor (TNFR) family and a soluble decoy receptor competitor of RANKL and soluble RANKL, which has been found to be a key factor in inhibiting osteoblast differentiation and activation [24, 25]. Furthermore, OPG expression increases osteoblast differentiation, whereas RANKL expression is inversely related to the degree of osteoblast differentiation [26]. Changes in OPG/RANKL levels play an important role in osteoblast and osteoclast homeostasis, and abnormal OPG/RANKL levels in the bone microenvironment can cause an imbalance in bone metabolism and eventually lead to a series of pathological changes in the skeletal system [27]. Osteoblasts are activated, and osteoclasts are restrained when OPG/RANKL expression is increased [28]. Moreover, increased OPG/RANKL levels can inhibit the apoptosis of tumour cells and play an important role in tumour development [29, 30]. In contrast to control cells, we observed clear increases in OPG expression levels, resulting in significantly increased OPG/RANKL levels, after miR-141-3p mimic transfection for 5 days (Figure 4A, 4B). These results further illustrate the miR-141-3p can induce high expression of OPG in osteoblasts, promote osteoblast activity and strengthen the interaction between cells, ultimately transforming the microenvironment of bone metastases. Next, we analysed the specific mechanism of miR-141-3p-induced OPG expression.

### Identification of miR-141-3p target genes

**Figure 5:**
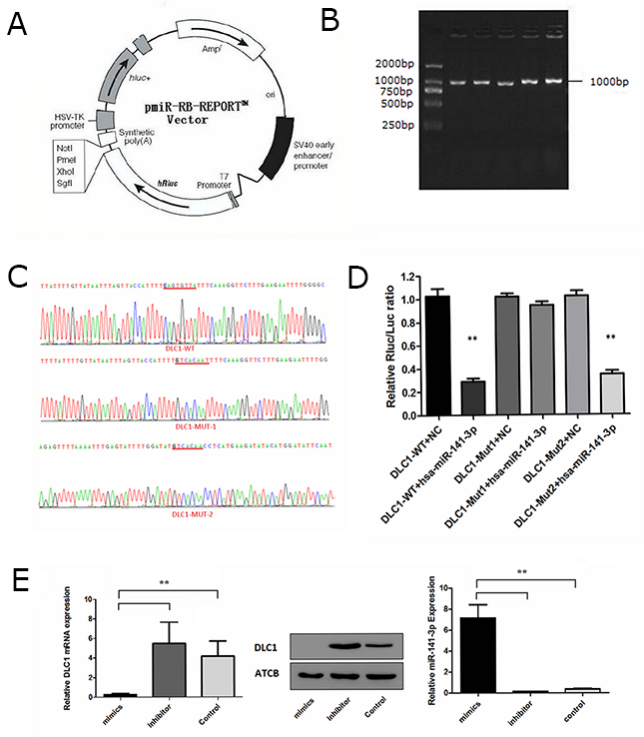
Identification of the miR-141-3p target gene **(A)** The pmiR-RB-REPORT™ dual-luciferase report vector. **(B)** Agarose gel electrophoresis of PCR products. **(C)** The results of targeted sequence. **(D)** Expression analysis of the dual-luciferase report gene. **(E)** The correlation between miR-141-3p expression and DLC1.

Target gene prediction analysis showed that DLC1 may be a target molecule of miR-141-3p. The 3’UTR of DLC1 was found to contain two putative miR-141-3p binding sites according to TargetScan 5.2 (http://www.targetscan.org/vert_50/) (Supplementary Figure 2). Primers were then designed based on the 3’UTR sequence of human DLC1 and used to PCR amplify the DLC1 3’UTR from 293T genomic DNA, which was cloned into the pmiR-RB-REPORT™ dual luciferase reporter plasmid (Figure 5A). Agarose gel electrophoresis yielded an amplification product of approximately 1000 bp, consistent with the predicted size (Figure 5B). Mutation primers were also designed based on the wild-type plasmid, and the target sequences CAGTGTTA and CAGTGTT were mutated to GTCACAAT and GTCACAA (Supplementary Table 2). All plasmids were sequenced and the results showed that target sequences were successfully mutated (Figure 5C). miRNA and the reporter gene plasmids were then transfected into cells, and the interaction between the miRNA and the target gene was verified based on the relative luminescence of the reporter. The expression analysis using the dual luciferase reporter system showed that miR-141-3p mimics lowered the luciferase activity of the DLC1-WT plasmid. Luminescence intensity recovered in the mutant plasmid in which the first predicted site (mut1) was disrupted, whereas the luminescence intensity of the mutant plasmid containing a mutation at the second predicted site (mut2) was obviously down-regulated (Figure 5D). We transfected osteoblasts with miR-141-3p mimics and inhibitor to alter the level of miR-141-3p in osteoblasts. Real-time PCR analysis showed miR-141-3p mimics/inhibitor can significantly change osteoblast miR-141-3p levels (*P* < 0.05). Simultaneously, miR-141-3p mimics significantly reduced the expression of DLC1, and the miR-141-3p inhibitor significantly increased it (*P* < 0.05) (Figure 5E). These results suggest that miR-141-3p negatively regulates DLC1 expression by binding the mut1 site.

### DLC1 inhibition by miR-141-3p is critical for activating the p38MAPK pathway

**Figure 6:**
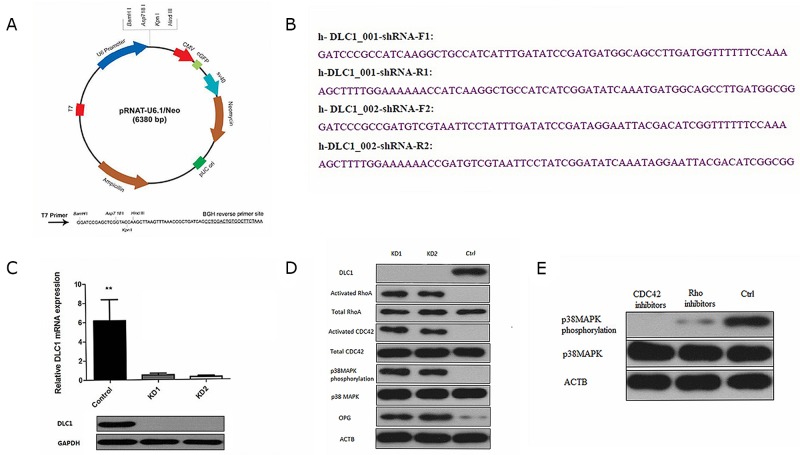
DLC1 inhibition is critical for mir-141-3p to activate the p38MAPK pathway **(A)** pRNAT-U6.1/Neo expression vector. **(B)** Two shRNA constructs and the corresponding primers. **(C)** Two shRNA constructs stably silence *DLC1* in osteoblasts. **(D)** *DLC1* KD activated RhoA and CDC42 and affected p38MAPK phosphorylation. **(E)** CDC42 inhibition significantly affected p38MAPK phosphorylation.

The DLC1 protein contains a highly conserved Rho-GAP domain that inhibits Rho-GTPases (specifically, RhoA and Cdc42) by promoting the hydrolysis of bound GTP to GDP and thus “shutting off” these proteins [31]. Rho-GTPases are involved in regulating cell morphology and migration, which can be involved in cancer-related signalling [32, 33]. Rho-GTPases are closely related to the activation of the p38MAPK pathway and play an important role in intercellular communication [34]. Therefore, we observed the effects of altering DLC1 expression on the p38MAPK pathway. We synthesized two shRNA constructs and knocked down DLC1 in osteoblasts to simulate the effect of miR-141-3p *in vitro* (Figure 6A, 6B and Supplementary Figure 3). Both shRNA constructs stably silenced DLC1 in osteoblasts with abundant DLC1 expression (Figure 6C). DLC1 KD activated RhoA and CDC42 but had no effect on total protein levels; GTPase activation by DLC1 KD also affected p38MAPK phosphorylation (Figure 6D). To analyse the specific mechanism of the osteoblast p38MAPK pathway, we observed changes in p38MAPK following treatment with Rho inhibitors (C3 transferase, Cytoskeleton, Inc.) and CDC42 inhibitors (ZCL278, ApexBio, USA) in DLC1 KD osteoblasts. The results showed that p38MAPK phosphorylation was significantly affected by CDC42 inhibition (Figure 6E).

### miR-141-3p-promoted osteoblast activity is p38MAPK dependent

**Figure 7:**
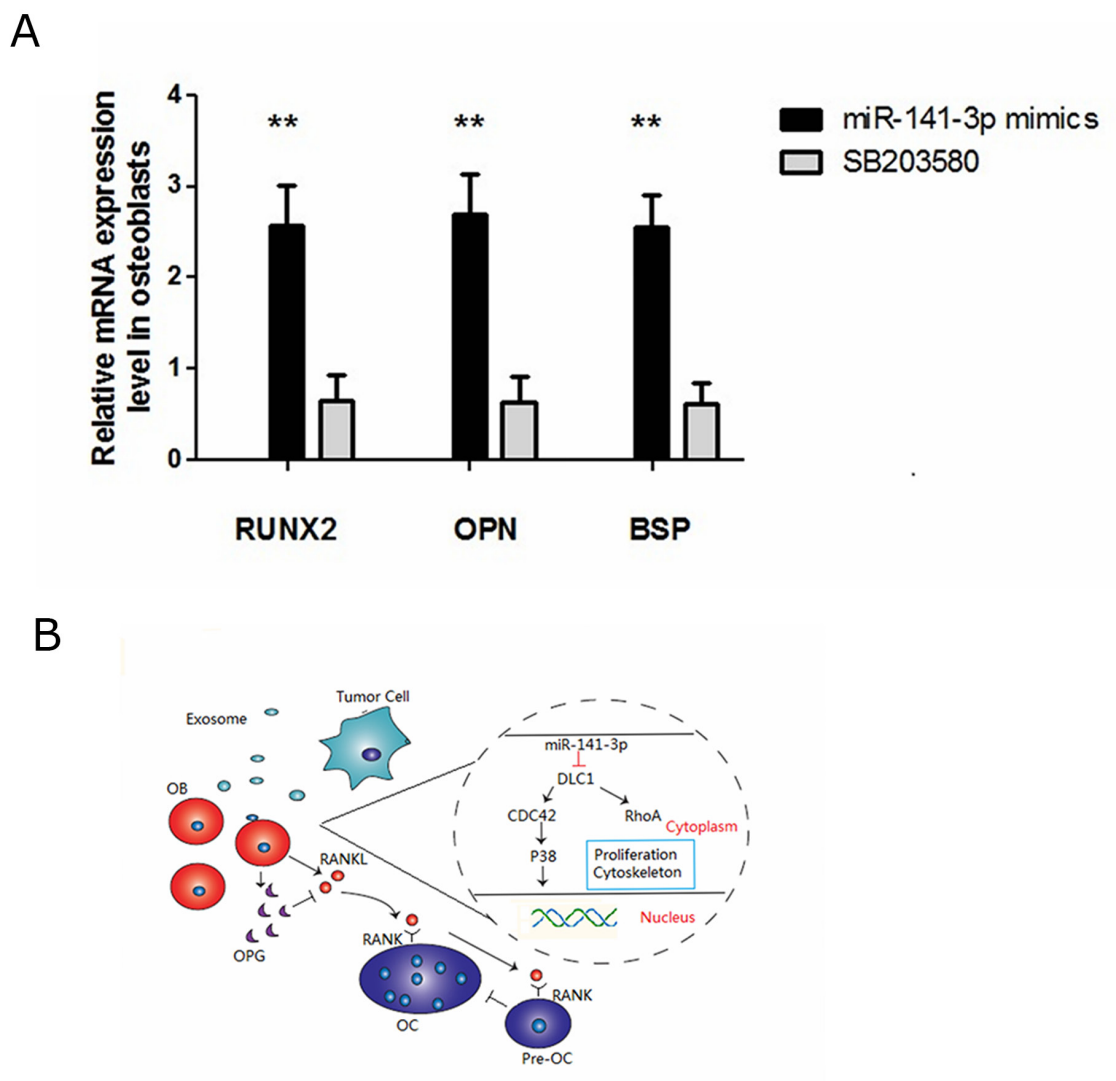
miR-141-3p-promoted osteoblast activity is p38MAPK dependent **(A)** Expression of osteoblast activity-related marker genes in osteoblasts. **(B)** DLC1-p38MAPK signalling in prostate cancer bone metastasis.

p38MAPK plays an important role in osteoblast activity, which can significantly increase alkaline phosphatase activity and calcium deposition [35]. Kusumi found that OPG expression was reduced, and RANKL expression was increased when p38MAPK signalling was blocked [36]. In the present study, we sought to confirm whether osteoblast activity was related to the activation of the p38MAPK signalling pathway. Therefore, we treated osteoblasts transfected with miR-141-3p mimics with a p38MAPK inhibitor (SB203580). Compared with the control cells, we found that the expression of osteoblast activity-related marker genes (Runx2, Opn and Bsp) in inhibitor-treated osteoblasts (SB203580) was significantly reduced (Figure 7A). OPG expression was also significantly reduced, which lead to decreased OPG/RANKL (Figure 4A, 4B). Above all, miR-141-3p activated p38MAPK signalling by inhibiting DLC1, promoted osteoblast activity and increased OPG/RANKL expression. These changes favour the formation of a bone-metastasis microenvironment and lay a foundation for the formation of PCa bone metastases (Figure 7B).

### Exosomal miR-141-3p promotes osteoblast activity *in vivo*

**Figure 8:**
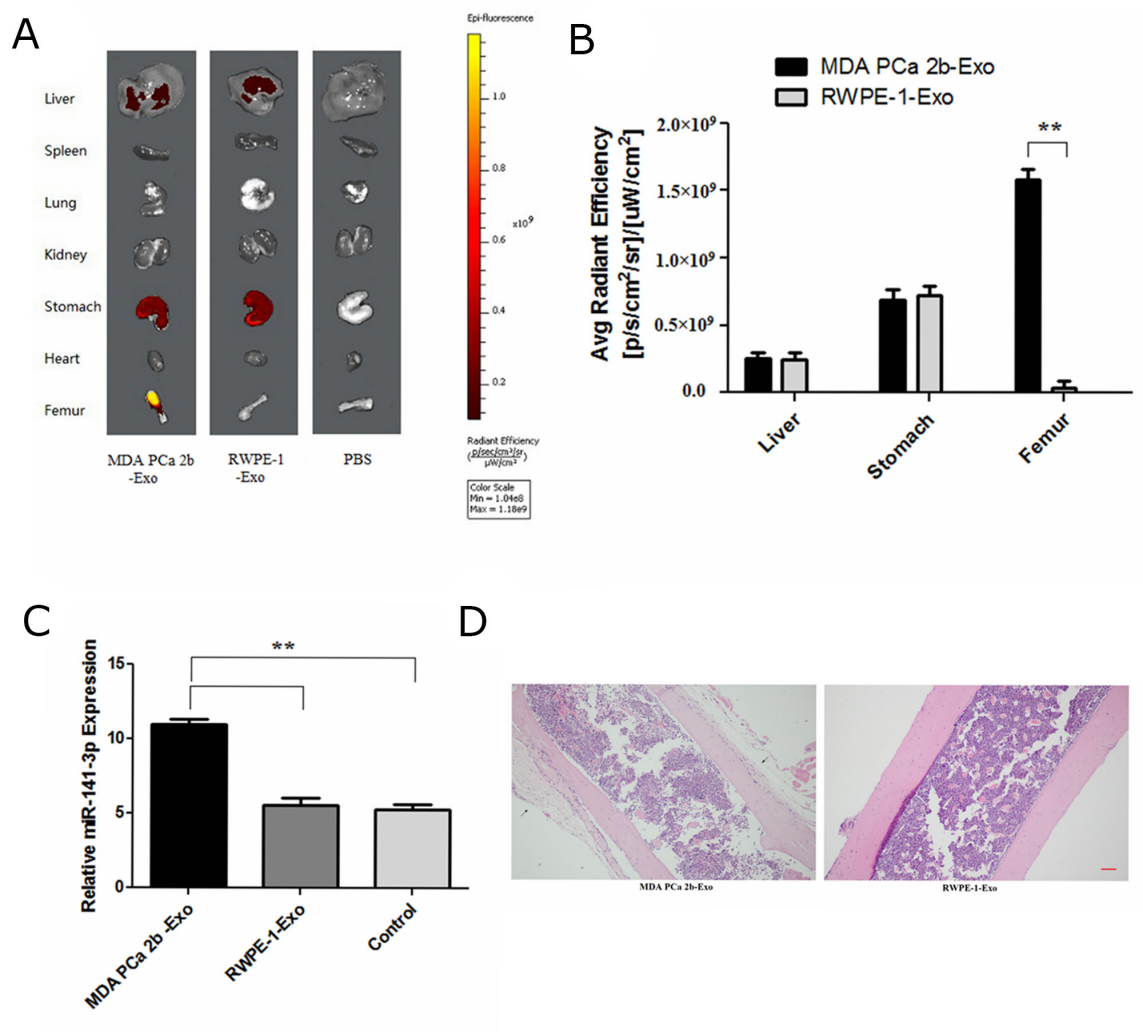
Exosomal miR-141-3p promotes osteoblast activity *in vivo* **(A)** Representative biophotonic images of the tissue distribution of fluorescence signal. **(B)** Average radiant efficiency of DiD-labeled exosomes. **(C)** The level of miR-141-3p in the distal femur of mice. **(D)** H&E staining of bone. Scale bars, 100 μm.

Then, we performed animal experiments to determine whether exosomal miR-141-3p from MDA PCa 2b cells promotes osteoblast activity *in vivo*. Six-week-old male *BALB/c nude mice* were intravenously injected with DiD-labelled exosomes (100 μg per mouse) isolated and purified from the supernatant of MDA PCa 2b or RWPE-1 cells; equal volume of phosphate-buffered solution (PBS) was used as a negative control. The distribution of DiD-exosomes was evaluated by biophotonic imaging at 8 h after injections. Fluorescence quantification determined significant accumulation of exosomes from MDA PCa 2b in the bone at 8 h after injections. Exosomes from MDA PCa 2b and RWPE-1 cells showed no significant differences at 8 h after injections, although the liver and stomach had fluorescent expression (Figure 8A, 8B). Six-week-old male *BALB/c nude mice* were intravenously injected with DiD-labelled exosomes (100 μg per mouse) from MDA PCa 2b or RWPE-1 cells, three times a week. At 4 weeks after the injections, real-time PCR analysis showed a significant increase of the level of miR-141-3p in the distal femur of mice treated with MDA PCa 2b exosomes (Figure 8C). HE staining of bone showed that a large number of osteoblasts were active in bone tissue of mice treated with MDA PCa 2b exosomes (Figure 8D).

### Exosomal miR-141-3p promotes osteoblastic metastasis

**Figure 9:**
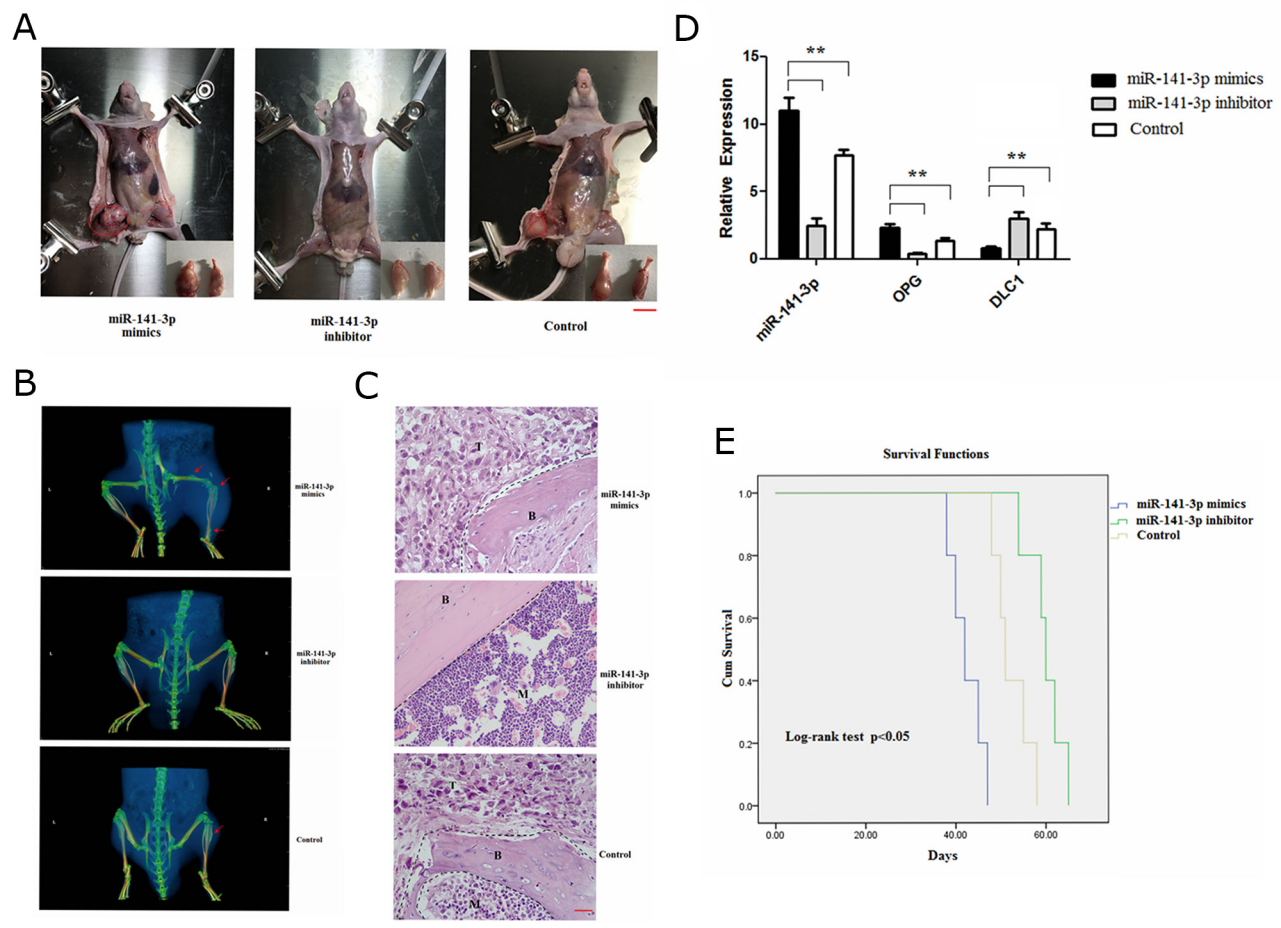
Exosomal miR-141-3p promotes osteoblastic metastasis **(A)** Mouse xenograft model. Scale bars, 1 cm. **(B)** Representative micro-CT images of bone metastases. Arrowheads denote areas of osteogenic changes. **(C)** H&E staining of bone metastases. B, bone; T, tumour; M, bone marrow. Scale bars, 50 μm. **(D)** Real-time PCR analysis of expression of miR-141-3p, DLC1, OPG. **(E)** Survival analysis.

We next examined whether exosomal miR-141-3p had the ability to promote the bone metastasis of PCa. We transfected MDA PCa 2b cells with miR-141-3p mimics/inhibitor. Six-week-old male *BALB/c nude mice* were divided into three group and respectively intravenously injected with exosomes from PCa cells transfected miR-141-3p mimics (A group), exosomes from PCa cells transfected miR-141-3p inhibitor (B group), and exosomes only from PCa cells (C group, control), three times a week (each time, 100 μg per mouse). At 3 weeks after the injections, the animals respectively received 2.0×10^6^MDA PCa 2b cells transfected with miR-141-3p mimics (A group)/inhibitor (B group) and only MDA PCa 2b cells (C group, control) via intraosseous injection. Mice injected with miR-141-3p mimics developed apparent bone metastases compared with those injected with the miR-141-3p inhibitor and the control at 4 weeks after injection (Figure 9A). Micro-CT showed that the right femur/tibia formed visible metastases in mice injected with miR-141-3p mimics, with increased shadow density on the bone background; the bone shape had no obvious change (Figure 9B). HE staining of bone showed osteogenic changes, and there was a large amount of osteoblasts and osteogenesis with tumour characteristics in the limbic cortex. The tumour cells were closely aligned, with loss of polarity and an imbalance of the nuclear to cytoplasmic ratio, and there was increased pathological nuclear fission (Figure 9C). There was a significant increase in the level of miR-141-3p and OPG and a significant decrease in DLC1 in the distal femur of mice treated with miR-141-3p-mimic exosomes (Figure 9D). The median survival times in the three groups (miR-141-3p mimics, miR-141-3p inhibitor and control) were 42, 60, 51, and the log-rank method test showed significant differences in the survival times of the three groups (*χ*2 = 9.701, *P* = 0.02). The mice injected with miR-141-3p mimics exhibited a significantly shorter survival time (Figure 9E). Collectively, these results indicate that exosomal miR-141-3p promotes osteoblastic metastasis *in vivo*.

## DISCUSSION

In recent years, the incidence of PCa has been increasing [37]. The occurrence of bone metastases is an important clinical feature and cause of death in PCa [38]. In PCa bone metastases, pure osteogenesis-type metastases accounts for approximately 95% of bone metastases [39]. However, the specific mechanism of PCa bone metastasis remains unclear [40]. A growing body of evidence supports the “seed and soil” model proposed by Paget, which posits a specific, strong interaction between PCa cells and the bone microenvironment [41]. The tumour microenvironment has become a major research focus in tumour biology and cancer medicine due to its potential to reveal the key mechanism of tumour metastasis [42]. Cancer-derived exosomes have been revealed to play an increasingly important role in microenvironment transformation [43, 44]. Cancer cells are reported to secrete a large number of exosomes, which are rich in miRNAs that can integrate into target cells and affect their physiological function [45, 46]. Most importantly, exosomes can enter the blood circulation and reach metastatic loci before cancer cells, thereby stimulating the microenvironment and promoting metastasis [47]. These results suggest that exosome-mediated miRNA transport plays an important role in cancer development.

Osteoblastic metastasis of PCa is characterized by a large degree of pathological osteogenesis, new bone that does not have the function of normal bone but instead destroys normal bone structure. The occurrence of osteoblastic metastasis is the result of interactions between PCa cells and osteoblast proliferation [18]. Osteoblasts are the key ingredient of the tumour microenvironment in bone metastases, playing an important role in influencing the osteoblastic metastasis of PCa [48]. In the bone-metastasis microenvironment, interactions among cancer cells, osteoblasts and osteoclasts promote tumour growth in bones and affect the normal balance of bone reconstruction, thereby promoting bone damage by tumour cells. Hence, we wanted to explore whether the miR-141-3p secreted by MDA PCa 2b cells is involved in the pathological process of bone rebuilding in osteoblastic metastasis and in the regulation of osteoblast activity.

We extracted exosomes from cell culture supernatants and identified them based on their morphology, size and marker protein expression. Compared with other PCa cell lines and RWPE-1 cells, in MDA PCa 2b cells, the levels of miR-141-3p increased significantly in exosomes. We then co-cultured either MDA PCa 2b cells (high expression of miR-141-3p) or RWPE-1 cells (low expression of miR-141-3p) with osteoblasts in a transwell system, observe the effect of miR-141-3p on osteoblasts activity by transfection of mimics. Numerous studies have detected significantly elevated serum miR-141-3p levels in PCa patients, which closely relate to tumour metastasis [13, 49]. Our results indicate that miR-141-3p can be transferred to osteoblasts through exosomes from metastatic PCa cells MDA PCa 2b *in vitro*. miR-141-3p promotes osteoblast activity to regulate the tumour microenvironment, which is beneficial for tumour growth and metastasis.

We then analysed the specific mechanism by which miR-141-3p regulates osteoblast activity. Accumulating evidence shows that miR-141-3p can inhibit the expression of DLC1, and the expression of these factors is negatively correlated [50, 51]. Dual-luciferase reporter assays revealed the targeted regulatory relationship between miR-141-3p and DLC1. Our results suggest that miR-141-3p negatively regulates DLC1 by interacting with a complementary site in the DLC1 mRNA. DLC1 is primarily involved in regulating the Rho GTPases (RhoA and Cdc42), which are involved in cancer-related signalling and are closely related to the occurrence and development of tumours [52]. Rho-GTPases act upstream of p38MAPK and activates the p38MAPK signalling pathway via the activation of MAP3Ks [53]. In the present study, we observed the effects of DLC1 down-regulation on the p38MAPK pathway. We further confirmed that the activation of the p38MAPK pathway is associated with CDC42 activation and that CDC42 activation is related to the targeted inhibition of DLC1 expression by MDA PCa 2b -derived exosomal miR-141-3p.

We then studied the effects of the p38MAPK pathway on osteoblast activity. P38MAPK activation increased the expression of OPG/RANKL, and treatment with a p38MAPK inhibitor (SB203580) had the opposite effect. We conclude that miR-141-3p effectively induces osteoblast differentiation and maturation and regulates the expression of OPG and RANKL by activating the p38MAPK pathway and suggest that miR-141-3p can stimulate osteoblastic activity, thereby resulting in pathological bone formation. Taken together, our findings delineate a new pathway regulating prostate-to-bone metastasis via exosomes (Figure 7B). By directly promoting osteoblast function and indirectly inhibiting osteoclasts, exosomal miR-141-3p from bone-metastatic PCa cells (MDA PCa 2b) promotes osteoblast activity and regulates the bone-metastasis microenvironment.

Next, we conducted an *in vivo* experiment to investigate whether exosomal miR-141-3p could be transferred to the bone and regulate osteoblast activity. In this study, fluorescent-tagged exosomes allowed direct *in vivo* visualization to determine its systemic biodistribution. Here, we report that exogenously administered (intravenous) exosomes from MDA PCa 2b distributed predominately to the bone, a frequent site of PCa metastasis. Exosomes from MDA PCa 2b cells also distributed to both the liver and stomach, but showed no significant difference from exosomes from RWPE-1 cells; this phenomenon was analysed for non-specific expression. Fluorescent-tagged exosomes and HE staining of bone showed that exosomal miR-141-3p from MDA PCa 2b cells had bone-target specificityand promoted osteoblast activity.

Considering the role of exosomal miR-141-3p derived from MDA PCa 2b cells in regulating osteoblasts activity, we performed animal experiments to evaluate whether exosomal miR-141-3p may promote osteoblastic metastasis. The results show that mice injected with miR-141-3p-mimic exosomes developed apparent osteoblastic bone metastasis at 4 weeks after injection and exhibited a significantly shorter survival time. In brief, we performed a series of studies *in vitro* and *in vivo* to identify that MDA PCa 2b cells -derived exosomal miR-141-3p transferred to osteoblasts and regulates its activity to promote osteoblastic metastasis and specific molecular mechanisms. miRNA-mediated cancer cell-to-osteoblast communication, which plays an important role in the formation of bone metastases and osteogenic damage in PCa. Further animal and clinical studies of the targeted inhibition of exosomal miR-141-3p expression may pave the way for the prevention and treatment of bone metastases in PCa, and further clarifying the specific mechanisms of bone metastasis will provide new ideas for the treatment of PCa.

## MATERIALS AND METHODS

### Cell culture

Human MDA PCa 2b, RWPE-1 and hFOB1.19 cells were obtained from ATCC (Manassas, VA, USA). MDA PCa 2b cells, RWPE-1 cells were cultured in F12K nutrient mixture supplemented with L-Glutamine (Gibco BRL Co. Ltd., USA), and hFOB1.19 cells were cultured in DMEM/F12 (HyClone, Logan, UT, USA); all culture media were supplemented with 100 units of penicillin/mL and 100 mg of streptomycin/mL (HyClone, Logan, UT, USA) and 10% FBS (Gibco BRL Co. Ltd., USA). The cells tested negative for mycoplasma contamination, and this testing was completed every 3 months and after the initiation of cell culture.

### Exosome isolation

Isolation of exosomes from cells was performed as previously described [54]. Briefly, cell lines were grown in FBS-supplemented culture media that was depleted of exosomes. Supernatant fractions were collected from 48-h cell cultures, followed by centrifugation (500 x *g*; 10 min) and filtration (0.22 μm, Millipore) to remove dead cells and large debris. Exosomes were collected, washed in PBS and pelleted by ultracentrifugation at 100,000 x g for 90 min at 4**°**C and finally resuspended in PBS. The size distribution of the exosomes was examined using a NanoSight Tracking Analysis LM20 System (NanoSight Ltd). EM imaging was performed on a TEM-1400plus transmission electron microscope. Exosome protein concentration was determined using the BCA protein assay (Fisher Scientific), and 20 μg/μl concentration of the exosome suspension was used for all experiments.

### Western blotting

Cells were harvested, and protein was extracted from cells as described previously [55]. Protein concentrations were determined using a protein assay kit (Bio-Rad, Hercules, CA, USA), and samples were separated via SDS polyacrylamide gel electrophoresis at various concentrations depending on the molecular weight of the protein under investigation. The blotting membrane was blocked with bovine serum albumin and incubated with rabbit monoclonal anti-CD63 antibody (1:1000; ab134045; Abcam), rabbit monoclonal anti-GM130 (1:1000; ab52649; Abcam), rabbit polyclonal anti-DLC1 antibody (1:1000; ab126257; Abcam), rabbit polyclonal Cdc42 antibody (1:1000; A13984; Thermo Fisher), rabbit polyclonal anti-P38 antibody (1:1000; ab27986; Abcam), rabbit phospho-p38 MAPK alpha polyclonal antibody (1:1000; 44-684G; Thermo Fisher), rabbit polyclonal anti-RhoA antibody (1:500; ab86297; Abcam) and RhoA/Rac1/Cdc42 Combo Activation Assay (1:1000; ab211168; Abcam), followed by incubation with horseradish peroxidase (HRP)-coupled goat anti-rabbit IgG H&L (1:5000; ab6721; Abcam) or HRP-coupled goat anti-mouse IgG H&L (1:5000; ab97040; Abcam). Primary antibodies were incubated overnight at 4°C using the dilutions. C3 transferase (1 μg/ml; 30 min; Cytoskeleton) was used as a Rho inhibitor; SB203580 (10 μM; 15 min; Gene Operation; USA) was used as p38 MAPK inhibitor; and ZCL278 was used as Cdc42 inhibitor (50 μM; 1 h; Selleck; USA).

### Co-culture and transfection experiments

Transwell inserts with a 0.4-mm pore-sized filter (Sigma Aldrich, St. Louis, MO, USA) for six-well plates were used following the manufacturer’s instructions. Osteoblast (5 × 10^4^ cells) cells were seeded into the lower chamber, and MDA PCa 2b (2.5 × 10^4^ cells) or RWPE-1 cells (5 × 10^4^ cells) were seeded into the top chamber cultured for 72 h in 4 ml of foetal bovine serum-containing medium (1:1 mix of F12k and DMEM/F12 supplemented with 100 units of penicillin/mL, 100 mg of streptomycin/ml and 10% FBS). A lentiviral vector system encoding cytomegalovirus (CMV)-driven RFP-tagged CD63 (CMV-RFP-CD63) to label the exosomes was purchased from Shanghai SunBio Biomedical Technology CO., LTD. MDA PCa 2b and RWPE-1 cells were transfected at a multiplicity of infection (MOI) of 100 on the day before to co-culture experiments according to the manufacturer’s instructions.

### Stress fibre staining

When cells reached 50% confluence, they were starved in DMEM containing 0.5% BSA. After 24 h, the cells were fixed with 10% formalin for 15 min, permeated with 0.1% Triton X-100 for 10 min, and stained with 5 U/ml rhodamine-phalloidin (Invitrogen) for 20 min. Afterwards, cells were washed twice with PBS and stained with DAPI (1 μg/ml) (Invitrogen). Stained cells were then imaged on a laser confocal microscope.

### RNA preparation and RT-PCR

Total RNA was isolated from cells or bone using TRIzol reagent (Life Technologies) and then treated with TURBO Dnase (Ambion). The total RNA was reverse transcribed into first-strand cDNA using a high-capacity cDNA reverse transcription kit (Takara) following the manufacturer’s instructions. To detect miR-141-3p expression levels, total RNA was isolated from exosomes using the mirVana miRNA Isolation Kit following the manufacturer’s instructions (Ambion). Exosomal RNA was reverse transcribed to cDNA with M-MLV Reverse Transcriptase System (Promega, USA) using 2 μl of 100 pM Random primers and 8 μl of exosomal RNA. The cDNA was diluted 1:4 and then quantified with SYBR Green Master (Rox) (Roche, USA). qRT-PCR was performed using FastStart Universal SYBR Green Master (Rox) on an ABI 7500 (Applied Biosystems). The amplification conditions were 95°C for 10 min, followed by 40 cycles of 95°C for 10 s and 60°C for 30 s. Expression values were normalized to GAPDH or U6, and the relative expression of the miRNAs was calculated by 2^-ΔΔCT^. The primers used for qRT-PCR were purchased from Shanghai Sangon Biotech Co., Ltd. (Supplementary Table 3).

### shRNA vector construction

We used PRNAT-U6.1/Neo as the interference in expression vector, diluted synthetic deoxyribonucleotide to 1 μg/μl, and combined the corresponding 5 μl each of the F and R chains to form double-stranded RNA by annealing. The shRNA expression vector pRNAT-U6.1/Neo was digested with BamHI and HindIII for 4 h at 37°C, and the product was gel-extracted and transformed into *E. coli*. The ligation product was added to 100 μl of DH5ɑ competent cells and placed in an ice-bath for 30 min. The tube was incubated at 42°C for 90 sec and then immediately put in an ice bath for 2-3 min. After adding 250-500 μl of LB culture medium preheated to 37°C to the tube, the culture was shaken at 150 rpm at 37°C for 45 min. Then, the bacterial culture was mixed, and 100 μl was transferred to the LB solid culture medium containing AMP antibiotics; the cells were smeared evenly using a sterile bend glass rod. A single colony was inoculated in 4 ml of LB culture medium containing Amp antibiotics. The cells were cultured at 37°C for 12-16 h with vigorous shaking. After overnight culture, the bacterium was extracted plasmid and sequenced, and the sequencing results showed that the fragment inserted correctly into the vector. All plasmids were sequenced by (GUANGZHOU RIBOBIO CO., LTD).

### Target gene identification

A total of 1.5×10^4^ exponentially growing 293T cells (low-passage) were inoculated in 96-well plates and incubated at 37°C for 24 h. miRNA mimics or non-target controls were diluted with 10 μl of OPTI-MEM medium; target gene 3 ’UTR double report gene carrier or mutation carriers were diluted with 15 μl of OPTI-MEM medium, and 0.25 μl of LipofectamineTM 2000 reagent was diluted with 25 μl of OPTI-MEM medium. The three were mixed after 5 min to obtain a total of 50 μl. After standing for 20 min, the transfection concentration was 50 nM, and the plasmid concentration was 250 ng/well. Reactions were initiated by adding 35 μl of luciferase substrate to each 48 h after transfection, followed by 30 μl of stop solution after 10 min. Luminescence was detected using a fluorescence light meter.

### Imaging of fluorescently labelled exosomes and tracking *in vivo*

Purified exosomes were fluorescently labelled using Vybrant® DiD (Life Technologies) according to manufacturer’s instructions with modifications. Briefly, exosomes were incubated for 20 min with DiD (1:1000 dilution in PBS). Excess dye was removed by washing in 20 ml of PBS at 100,000 x g (90 min) to receive the final DiD stained exosome preparation. DiD-labeled exosomes derived from MDA PCa 2b or RWPE-1 cells were injected intravenously into 6-week-old male BALB/C mice (100 μg of exosomes/mouse). Thereafter, the mice were sacrificed at 8 h after the injection, and various tissues (lung, spleen, kidney, stomach, liver, heart and femur) were harvested for biophotonic imaging. Intensity of fluorescence was quantified using the IVIS Lumina II® Spectrum and Living Image® 4.3.1 Software (PerkinElmer) to assess tissue distribution of DiD-labeled exosomes.

### Tumorigenesis studies of animal

All experimental procedures were approved by the Committees of Animal Ethics and Experimental Safety of Fourth Military Medical University. To analyse tumourigenesis, 6-week-old male nude mice were injected by intraosseous with 2.0×10^6^ tumour cells in 50% Matrigel™ (Falcon, NJ, USA). For survival studies, mice were euthanized when one of the following situations applied: 10% loss of body weight, paralysis, or head tilting. All animal studies were repeated three times.

### Micro-CT analysis

Micro-CT analysis was performed using a *Siemens Inveon Micro-CT* (*Siemens*, Germany) equipped with an X-ray tube (voltage, 80kV; current, 500 μA), and the pixel size was set to 47.77 μm.

### Statistical analyses

Unless otherwise indicated, the data are presented as the mean ± SD. Non-parametric data were analysed by 2-tailed Mann-Whitney U-tests. Parametric data were analysed using ANOVA with post hoc comparison (Tukey method). Kaplan-Meier was adopted for survival analysis and to generate the survival curve; log-rank was used to test the significance of differences between the three groups. An adjusted *P*-value < 0.05 was considered significant. All statistical analyses were performed with SPSS software at version 18.0 (SPSS Inc.).

## SUPPLEMENTARY MATERIALS FIGURES AND TABLES


