# Supplementary material for: Exosomal miR-141-3p regulates osteoblast activity to promote the osteoblastic metastasis of prostate cancer
Source: Oncotarget. 2017 Oct 24;8(55):94834–49. doi: 10.18632/oncotarget.22014 (PMC5706916; doi:10.18632/oncotarget.22014)
Supplement: Supplementary file 1 [file oncotarget-08-94834-s001.pdf]

Exosomal miR-141-3p regulates osteoblast activity to promote the osteoblastic metastasis of prostate cancer

SUPPLEMENTARY MATERIALS

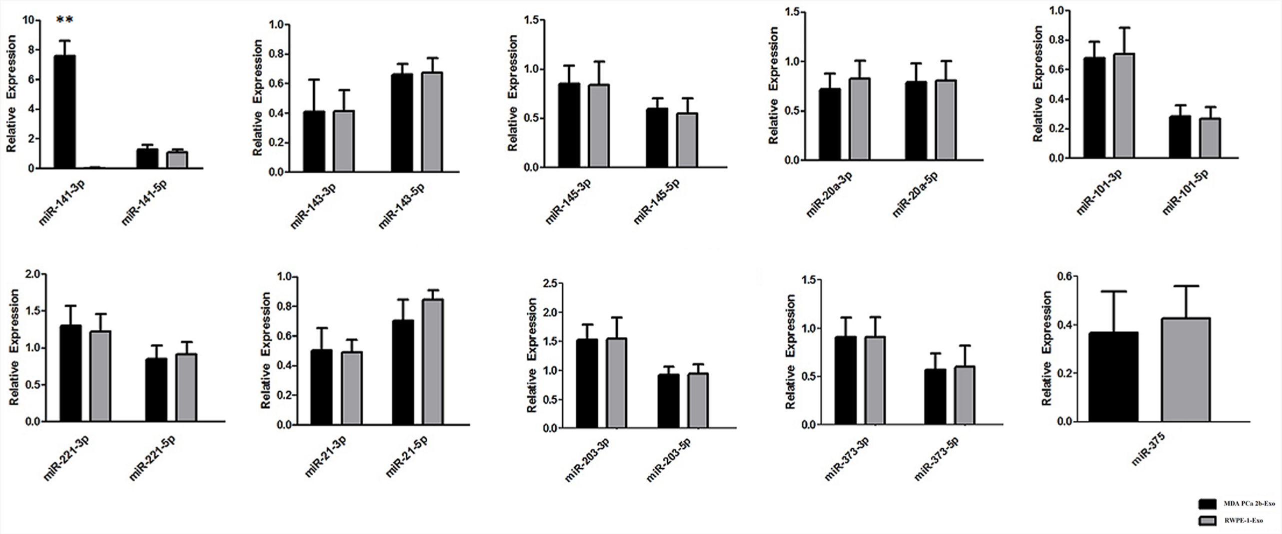

Supplementary Figure 1: Real-time PCR analysis of 10 miRNAs involved in bone metastases in exosomes from cell supernatants.

	Predicted consequential pairing of target region (top) and miRNA (bottom)	Site type	Context++ score	Context++ score percentile	Weighted context++ score	Conserved branch length	P_{CT}
Position 1467-1474 of DLC1 3' UTR<br>hsa-miR-141-3p	5' ...UUUAGUUACCAUUUUCAGUGUUA... |||||| ||||||| 3' GGUAGAAAUGGUCU--GUCACAAU	8mer	-0.37	99	-0.28	4.194	0.71
Position 1548-1554 of DLC1 3' UTR<br>hsa-miR-141-3p	5' ...GAGUAUUUUGGAUAUCAGUGUUC... ||||||| 3' GGUAGAAAUGGUCUGUCACAAU	7mer-m8	-0.13	85	-0.09	4.387	0.46

Supplementary Figure 2: miR-141-3p target gene prediction.

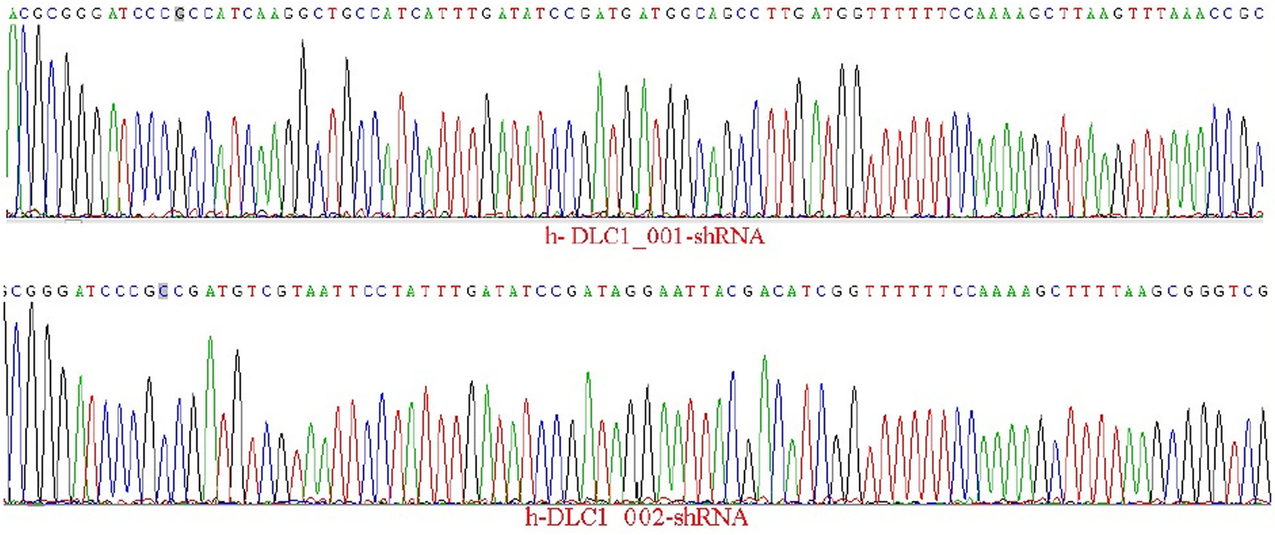

Supplementary Figure 3: The results of shRNA sequencing: correct insertion of the targeted fragment into the vector.

Supplementary Table 1: Ten miRNAs examined exosomes from cell supernatant

hsa-miR-141-5p	MIMAT0004598	CAUCUUCCAGUACAGUGUUGGA
hsa-miR-141-3p	MIMAT0000432	UAACACUGUCUGGUAAAGAUGG
hsa-miR-20a-5p	MIMAT0000075	UAAAGUGCUUAUAGUGCAGGUAG
hsa-miR-20a-3p	MIMAT0004493	ACUGCAUUAUGAGCACUUAAAG
hsa-miR-145-5p	MIMAT0000437	GUCCAGUUUUCCCAGGAAUCCCU
hsa-miR-145-3p	MIMAT0004601	GGAUUCCUGGAAAUACUGUUCU
hsa-miR-143-5p	MIMAT0004599	GGUGCAGUGCUGCAUCUCUGGU
hsa-miR-143-3p	MIMAT0000435	UGAGAUGAAGCACUGUAGCUC
hsa-miR-101-5p	MIMAT0004513	CAGUUAUCACAGUGCUGAUGCU
hsa-miR-101-3p	MIMAT0000099	UACAGUACUGUGAUAACUGAA
hsa-miR-221-5p	MIMAT0004568	ACCUGGCAUACAAUGUAGAUUU
hsa-miR-221-3p	MIMAT0000278	AGCUACAUUGUCUGCUGGGUUUC
hsa-miR-375	MIMAT0000728	UUUGUUCGUUCGGCUCGCGUGA
hsa-miR-21-5p	MIMAT0000076	UAGCUUAUCAGACUGAUGUUGA
hsa-miR-21-3p	MIMAT0004494	CAACACCAGUCGAUGGGCUGU
hsa-miR-203a-5p	MIMAT0031890	AGUGGUUCUUAACAGUUCAACAGUU
hsa-miR-203a-3p	MIMAT0000264	GUGAAAUGUUUAGGACCACUAG
hsa-miR-373-5p	MIMAT0000725	ACUCAAAAUGGGGGCGCUUUCC
hsa-miR-373-3p	MIMAT0000726	GAAGUGCUUCGAUUUUGGGGUGU

Supplementary Table 2: Primers used for identification of miR-141-3p target genes

h_DLC1_3UTR_F	GCGGCTCGAGCATTGGGCTTGTTCTTATC
h_DLC1_3UTR_R	AATGCGGCCGCCAGACTTTTCCCTTACTTT
h_DLC1_mut1_F	ACCATTTTGTCACAATTTTCAAAGGTTCTTTGAA
h_DLC1_mut1_R	CTTTGAAAATTGTGACTAATTTAGTTACCATTTT
h_DLC1_mut2_F	TTGGATATGTCACAACCTCATGAAGATATACAT
h_DLC1_mut2_R	TCATGAGGTTGTGACATATCCAAAATACTCAAA

Supplementary Table 3: Primers used for real-time PCR

**NM_004967.3**	**Primer Set 1: Amplicon Size = 129**
BSP	GGCACCAGTACCAACAGCAC
BSP	CTGCCTTCCGGTCTCTGTGG
NM_000582.2	Primer Set 1: Amplicon Size = 105
OPN	CTGGGAGGGCTTGGTTGTCA
OPN	GTCGGCGTTTGGCTGAGAAG
NM_001015051.3	Primer Set 1: Amplicon Size = 186
Runx2	TGAGCTCCGGAATGCCTCTG
Runx2	CTGGGTTCCCGAGGTCCATC
NM_002546.3	Primer Set 1: Amplicon Size = 117
OPG	AGTGCAATCGCACCCACAAC
OPG	TTCCAGCTTGCACCACTCCA
NM_003701.3	Primer Set 1: Amplicon Size = 95
RANKL	GGAGGCCGAGGCTATTCTCC
RANKL	AGGCGCAGTGGTTCACTTCT
NM_001164271.1	Primer Set 1: Amplicon Size = 146
DLC1	TGGACAACGACCGAACCACA
DLC1	CCATCTCAGTCGGTGCCTGT
NM_001256799.2	Primer Set 1: Amplicon Size = 136
GAPDH	GGGTGTGAACCATGAGAAGT
GAPDH	GACTGTGGTCATGAGTCCT
miR-141-3p RT	GTCGTATCCAGTGCAGGGTCCGAGGTATTC GCACTGGATACGACCCATCTTT
miR-141-3p	ATGGTTCGTGCGTAACACTGTCTGGTAAA
miR-141-5p RT	GTCGTATCCAGTGCAGGGTCCGAGGTATTCG CACTGGATACGACTCCAACAC
miR-141-5p	ATGGTTCGTGCGCATCTTCCAGTACAGTG
U6 HoMsRn F	GCTTCGGCAGCACATATACTAAAAT
U6 HoMsRn R	CGCTTCACGAATTTGCGTGTCAT
HoMsRn R	GCAGGGTCCGAGGTATTC
